# Intercalation of Si between MoS_2_ layers

**DOI:** 10.3762/bjnano.8.196

**Published:** 2017-09-19

**Authors:** Rik van Bremen, Qirong Yao, Soumya Banerjee, Deniz Cakir, Nuri Oncel, Harold J W Zandvliet

**Affiliations:** 1 Physics of Interfaces and Nanomaterials, MESA+ Institute for Nanotechnology, University of Twente, P.O. Box 217, 7500AE Enschede, Netherlands; 2 Department of Physics and Astrophysics, University of North Dakota, Grand Forks, ND 58202, USA

**Keywords:** intercalation, molybdenum disulfide, scanning tunneling microscopy, silicene, two-dimensional materials

## Abstract

We report a combined experimental and theoretical study of the growth of sub-monolayer amounts of silicon (Si) on molybdenum disulfide (MoS_2_). At room temperature and low deposition rates we have found compelling evidence that the deposited Si atoms intercalate between the MoS_2_ layers. Our evidence relies on several experimental observations: (1) Upon the deposition of Si on pristine MoS_2_ the morphology of the surface transforms from a smooth surface to a hill-and-valley surface. The lattice constant of the hill-and-valley structure amounts to 3.16 Å, which is exactly the lattice constant of pristine MoS_2_. (2) The transitions from hills to valleys are not abrupt, as one would expect for epitaxial islands growing on-top of a substrate, but very gradual. (3) *I*(*V*) scanning tunneling spectroscopy spectra recorded at the hills and valleys reveal no noteworthy differences. (4) Spatial maps of d*I/*d*z* reveal that the surface exhibits a uniform work function and a lattice constant of 3.16 Å. (5) X-ray photo-electron spectroscopy measurements reveal that sputtering of the MoS_2_/Si substrate does not lead to a decrease, but an increase of the relative Si signal. Based on these experimental observations we have to conclude that deposited Si atoms do not reside on the MoS_2_ surface, but rather intercalate between the MoS_2_ layers. Our conclusion that Si intercalates upon the deposition on MoS_2_ is at variance with the interpretation by Chiappe et al. (*Adv. Mater.* **2014**, *26*, 2096–2101) that silicon forms a highly strained epitaxial layer on MoS_2_. Finally, density functional theory calculations indicate that silicene clusters encapsulated by MoS_2_ are stable.

## Introduction

Since the discovery of graphene [1–4] interest has extended to the search for other 2D materials with properties similar to graphene. One appealing candidate is silicene, a graphene-like 2D allotrope of silicon. The first calculations of graphite-like allotropes of silicon and germanium were performed by Takeda and Shiraishi in 1994 [5]. These authors pointed out that two-dimensional silicon and germanium are not planar but buckled, i.e., the two sub-lattices of the honeycomb lattice are displaced with respect to each other in a direction normal to the two-dimensional sheet. In addition, the calculations of Takeda and Shiraishi [5] also revealed that silicene and germanene are semi-metals, like graphene. In 2007, Guzmán-Verri and Lew Yan Voon [6] performed tight-binding calculations of two-dimensional silicon. They pointed out that the graphite-like silicon sheet has linearly dispersing energy bands near the *K* points of the Brillouin zone, very comparable to graphene. Inspired by this analogy they put forward the name silicene for the two-dimensional silicon. Interestingly, the linear dispersing energy bands at the *K* points, the so-called Dirac cones, are robust against the buckling of the silicene lattice [5,7]. In 2009, Cahangirov et al. [7] found that germanene also exhibits similar properties as graphene and silicene.

Similar to graphene, the electrons near the Fermi level in free-standing silicene are predicted to behave as massless Dirac fermions [6]. The broken sub-lattice symmetry of silicene allows for the opening of a band gap in this material [8–12]. This band gap makes silicene a very appealing candidate for field-effect-based devices. Another attractive property of silicene is its spin–orbit coupling, which is substantially larger than the spin–orbit coupling in graphene [13–14].

Silicene does not occur in nature and therefore it has to be synthesized. Several studies have reported on the growth of a 2D silicon layer on Ag(111) [15–17]. Unfortunately, due to the strong coupling between Si ad-layer and Ag substrate, the interesting Dirac properties of silicene are destroyed [18]. Although a linear dispersion relation has been observed [17], it is argued by others that this band is related to the Ag substrate rather than to silicene [19] or to combined effects of silicene and the Ag(111) substrate [20–21]. Growth of silicon was also demonstrated on graphite, a van der Waals material, with the idea to suppress the interaction with the substrate and as such to preserve the Dirac properties [22]. Unfortunately, graphite is metallic, which could also affect the electronic bands of silicene in the vicinity of the Fermi level. Van der Waals materials with a band gap do not suffer from this limitation. Molybdenum disulfide (MoS_2_) is a member of the transition metal dichalcogenide (TMD) family that belongs to the class of van der Waals materials. Bulk MoS_2_ has a band gap of 1.29 eV, which increases to 1.90 eV for a monolayer of MoS_2_ [23]. This means that MoS_2_ has no states near the Fermi level and therefore hybridization with the energy bands of silicene near the Fermi level cannot occur. Recently, germanene, a 2D allotrope of germanium [24–28], has already been successfully grown on MoS_2_ [29]. Chiappe et al. [30] deposited Si on MoS_2_ and found that Si forms an epitaxially strained layer on top of MoS_2_ with a lattice constant identical to the MoS_2_ lattice constant, i.e., 3.16 Å. A study confirming the two-dimensionality of deposited Si on MoS_2_ has recently been carried out using variable-angle X-ray photoelectron spectroscopy (XPS) [31]. It should be pointed out here that this study showed that the S 2p_3/2_ peak in MoS_2_ is at around 167.6 eV, which is considerably higher than the pure core-level line of pure S. This high value might be an indication of contamination with O [32] or Ni [33].

Here we revisit the growth of Si on MoS_2_. Our scanning tunneling microscopy (STM) observations are very similar to those reported by Chiappe et al. [30]. However, we arrive at the conclusion that Si intercalates between the MoS_2_ layers. In order to verify our conclusion we have performed additional spectroscopic measurements. These additional spectroscopic measurements unambiguously reveal that sub-monolayer amounts of Si deposited on MoS_2_ at room temperature do not reside on top of MoS_2_, but intercalate between the MoS_2_ layers.

## Experimental

The scanning tunneling microscopy and spectroscopy measurements were performed with an Omicron STM-1 room-temperature scanning tunneling microscope in ultra-high vacuum (UHV). The UHV system is composed of three separate chambers: a load-lock chamber for a quick entry of new samples and STM tips, a preparation chamber with facilities for sample heating, ion bombardment and evaporation of silicon and an STM chamber. The base pressures in the STM chamber and the preparation chamber are below 3 × 10^−11^ mbar and 5 × 10^−11^ mbar, respectively. The MoS_2_ samples are purchased from HQ graphene. Prior to inserting the samples into the lock-load system they were cleaned by mechanical exfoliation. Silicon was deposited on the MoS_2_ samples using a custom-built Si evaporator, which consists of a small piece of a Si wafer that can be heated resistively. The distance between substrate and evaporator is about 10 cm. The silicon was deposited at a rather low deposition rate of 0.8 nm·h^−1^. The silicon evaporator was calibrated by depositing a sub-monolayer amount of Si on a Ge(001) substrate. The Ge(001) surface was cleaned by applying several cycles of Ar ion sputtering and annealing. After deposition and mild annealing at a temperature of 450–500 K, the Ge(001) substrate was inserted into the STM and subsequently the areal coverage of the epitaxial Si islands was determined. *I*(*V*) curves are recorded at constant height at 450 ms per curve. Spatial maps of d*I*/d*z* are measured using a lock-in amplifier. A small high frequency (ca. 1.9 kHz) sinusoidal signal is added to the *z*-piezo and the tunnel current is fed into the lock-in amplifier. The output signal of the lock-in amplifier, which is proportional to d*I*/d*z*, is measured simultaneously with the topography.

MoS_2_ samples used for the X-ray photoelectron spectroscopy (XPS) experiments were purchased from nanoScience Instruments. The MoS_2_ samples were exfoliated before Si deposition. In a separate UHV chamber, Si was deposited on the MoS_2_ sample via resistive heating of a small piece of a silicon wafer and then the MoS_2_ sample was quickly transferred to the XPS chamber. During this transfer the sample was exposed to ambient conditions. The deposited amount of Si was 0.5 monolayers. The base pressure of both chambers is below 4 × 10^−10^ mbar. Both MoS_2_ and Si/MoS_2_ samples were measured with a monochromatic Al Kα (1486.6 eV) X-ray source with a pass energy of 89.5 eV and 35.75 eV for survey and high-resolution scans, respectively. During the XPS measurements, the pressure was kept at or below 1 × 10^−9^ mbar. The angle between the X-ray source, which is aligned along the surface normal, and spectrometer is 54.7°. All XPS core-level spectra were analyzed using Augerscan software, which is equipped with its own curve-fitting program. The core-level peaks are fitted using a Gaussian–Lorentzian (GL) function to include the instrumental response function along with the core-level line shape. The secondary-electron background was subtracted using a Shirley function [34]. The energy differences between the 3d and 2p spin–orbit couples were set to 3.13 eV and 1.18 eV, respectively. The ratios of the areas of the doublet peaks were also fixed. During sputtering the pressure is increased to 3 × 10^−8^ mbar by leaking in Ar gas while the pressure around the filament in the differentially pumped argon gas chamber increased to 1 × 10^−4^ mbar. The sample was sputtered with a beam of Ar ions with 1 kV energy. The emission current used was 25 mA, which resulted in an ion current of 0.33 μA. The shape of the beam is circular with a diameter of approximately 2 mm.

First-principles calculations are based on the projector-augmented wave (PAW) method [35–36] within DFT as implemented in the Vienna ab initio simulation package (VASP) [37]. The exchange–correlation interactions are treated using the generalized gradient approximation (GGA) within the Perdew–Burke–Ernzerhof (PBE) formulation [38]. The plane waves are expanded with an energy cut of 400 eV. Since the semi-local functionals, such as GGA, fail to describe weakly interacting systems, we also take into account the van der Waals interaction [39–40]. Brillouin-zone integrations for structure relaxations are approximated by using the special *k*-point sampling of the Monkhorst–Pack scheme with a Γ-centered 3 × 3 × 1 grid [41]. In order to minimize the periodic interactions along the *z*-direction (the direction perpendicular to the plane of the hetero-trilayer) the vacuum space between the layers has a width of at least 15 Å.

## Results and Discussion

**Figure 1:**
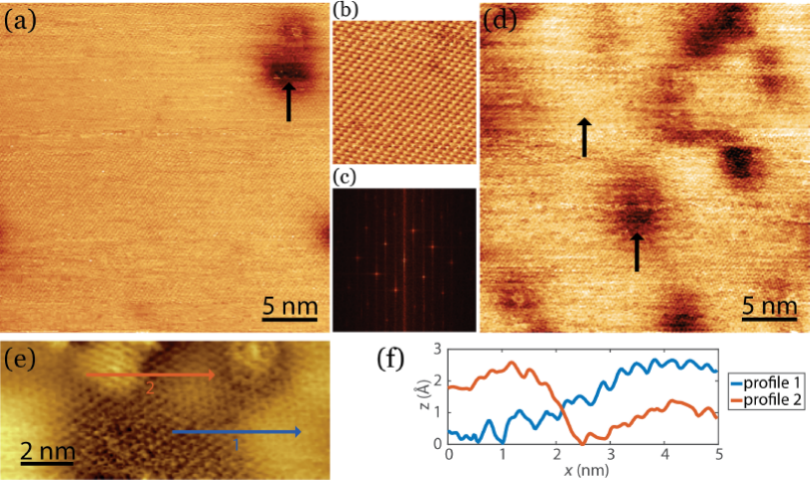
(a) STM image of pristine MoS_2_ taken prior to the deposition of Si. The arrow indicates an intrinsic defect, which is often found on MoS_2_. (b) High-resolution STM image of pristine MoS_2_. (c) Fast Fourier-transform of pristine MoS_2_ showing the hexagonal symmetry. (d) STM image taken after the deposition of 0.2 monolayers of Si. The arrows indicate a hill (bright) and a valley (dark). (e) High-resolution STM image taken after the deposition of 0.2 monolayers of Si. (f) Line scans taken along the lines indicated in panel (e). The sample bias is 1.2V and the tunnelling current is 0.5 nA.

In Figure 1, STM images of pristine MoS_2_ and MoS_2_ after the deposition of ca. 0.2 monolayers of Si at room temperature are shown. The pristine MoS_2_ surface appears very smooth. Usually only the top sulfur layer is resolved, resulting in a lattice with hexagonal symmetry and a lattice constant of 3.16 Å (see Figure 1b,c). The pristine MoS_2_ contains some intrinsic defects, which are visible as dark depressions as indicated by the arrow in Figure 1a. These defects are most probably caused by vacancies or interstitials and have been found to exhibit a metal-like behavior [42–43]. Upon the deposition of 0.2 monolayers of Si, the surface morphology converts to a hill-and-valley structure as shown in Figure 1d. The arrows indicate a bright hill and a dark valley. Upon further deposition of silicon, the surface becomes rougher and more difficult to scan as shown in Figure S1 in Supporting Information File 1. When even more silicon is deposited, silicon clusters on top of MoS_2_ become visible. A close-up image of the transition of a hill to a valley is represented in Figure 1e. The line profiles indicated in the figure correspond to the cross sections shown in Figure 1f. The typical height variation of a transition is found to be of several angstroms. We found a similar height variation using density functional theory (DFT) calculations of the intercalation of a single silicon layer in between two MoS_2_ layers. These calculations are discussed after the presentation of the experimental results. It is immediately obvious from Figure 1f that the transition from a hill to a valley is very gradual. Interestingly, the lattice constant of the hill-and-valley structure is identical to the lattice constant of pristine MoS_2_, i.e. 3.16 Å. Both observations are similar to the observations reported by Chiappe et al. [30] who deposited 0.8 monolayers of silicon on MoS_2_ (obtained from SPI) at 200 °C. Based on these observations Chiappe et al. [30] concluded that Si grows epitaxially on MoS_2_ with a lattice constant that is identical to MoS_2_. This implies that the Si layer is highly strained, indicative of a rather strong interaction between MoS_2_ and Si. This seems unlikely, bearing in mind that MoS_2_ is a van der Waals material. We tentatively put forward another interpretation, namely that Si intercalates between the MoS_2_ layers. The gradual transition from a hill to a valley as well as the observation of the MoS_2_ lattice constant after Si deposition nicely fits into this picture.

**Figure 2:**
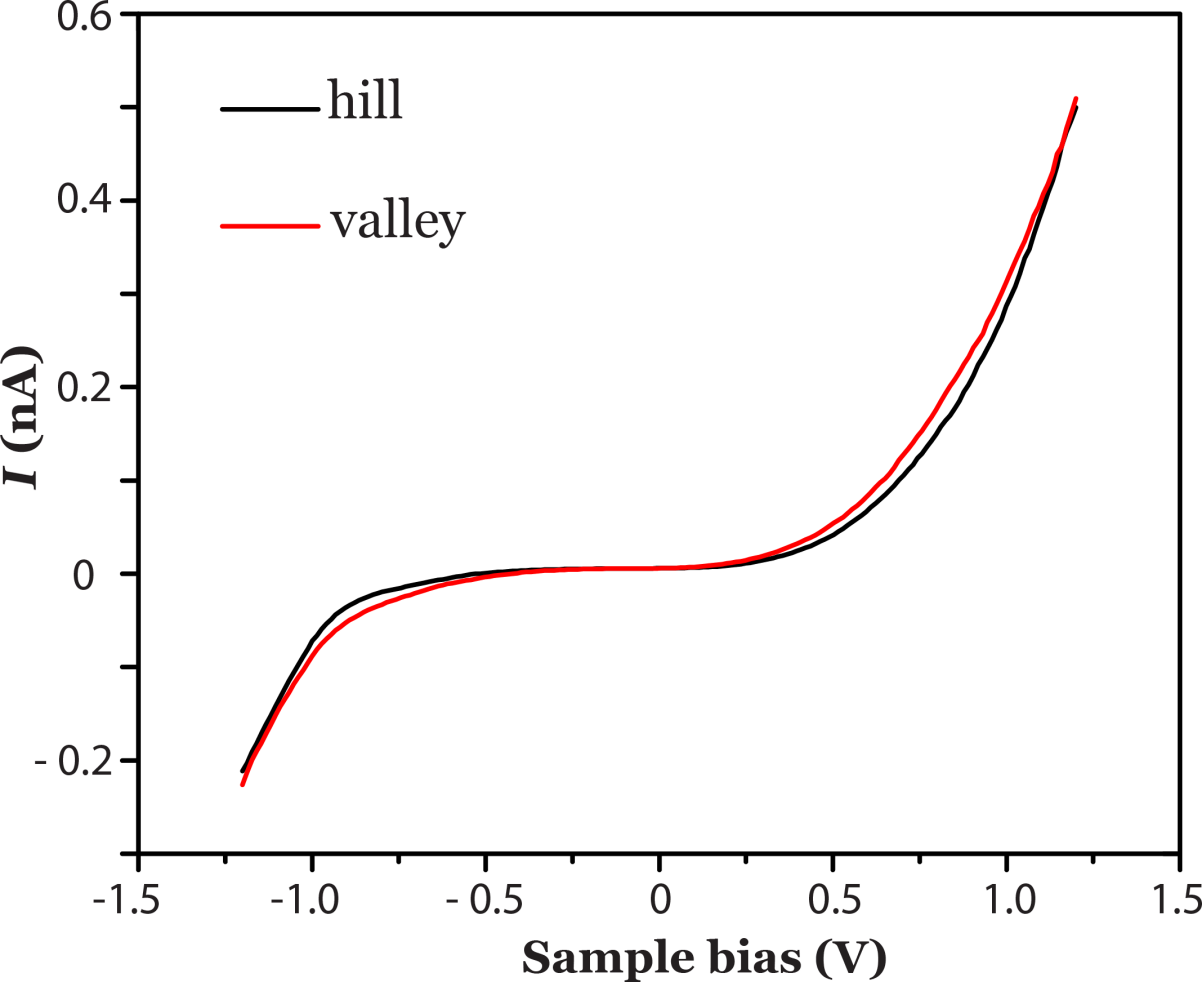
STS recorded at the hills (black curve) and at the valleys (red curve). Set points sample bias 1.2 V and tunnel current 0.5 nA.

In order to verify our interpretation we have performed additional scanning tunneling spectroscopy (STS) measurements. *I*(*V*) scanning tunneling spectra were recorded at the hills and valleys as indicated by the arrows in Figure 1d. Average spectroscopy curves of a hill and of a valley, which in total are comprised of 3500 spectra, are displayed in Figure 2. The *I*(*V*) spectra are almost identical to each other. The small difference between both curves might be a residual effect of Si residing underneath the MoS_2_ layer. If the top layer were a silicon cluster the *I*(*V*) spectra would differ significantly as is shown in Figure S2 in Supporting Information File 1.

**Figure 3:**
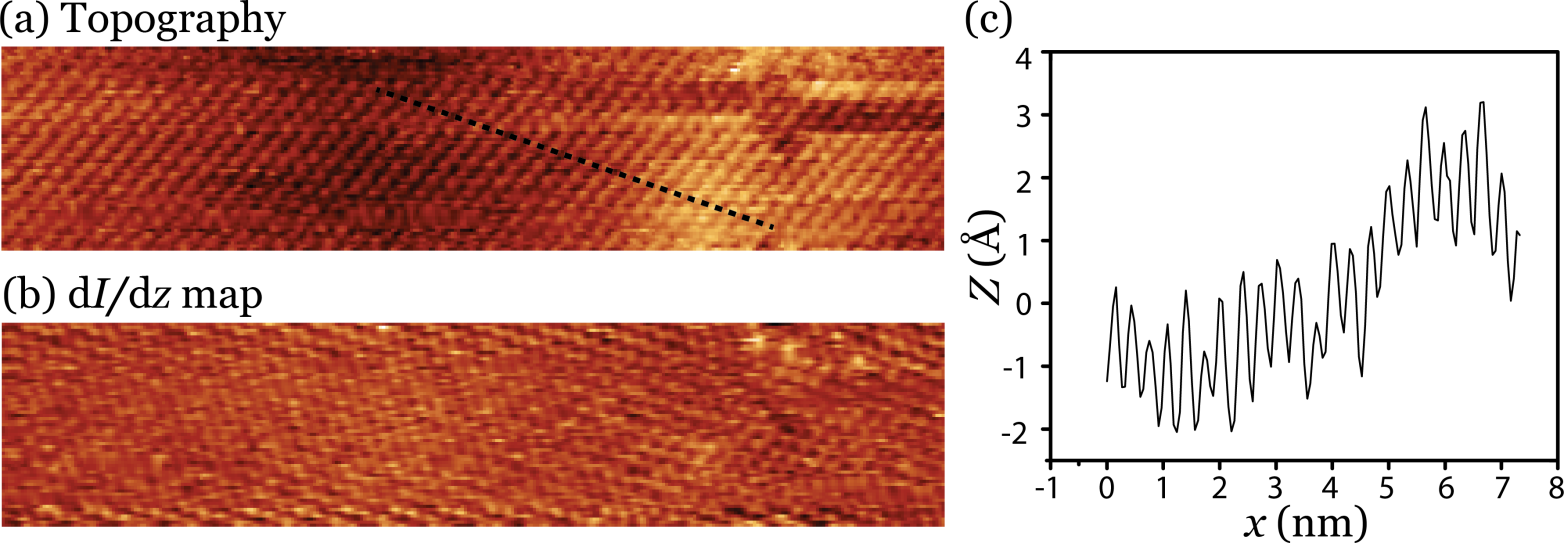
(a) STM image of a MoS_2_ surface after the deposition of 0.2 monolayers of Si. (b) Spatial map of d*I*/d*z*. In both images the atomic structure is resolved. (c) Line- scan taken along the dotted line depicted in panel (a). Sample bias is 1.2 V and tunnel current is 0.5 nA.

In order to remove the large-scale height variation from the topography scan, we simultaneously recorded a spatial map of d*I*/d*z* (Figure 3). The d*I*/d*z* signal only depends on the effective work function, also referred to as the apparent barrier height, and not on any large-scale height variations [44–45]. It should be pointed out here that spatial maps of d*I*/d*z* often exhibit a resolution that is similar to normal topographic STM images without, of course, the large-scale height variations [44].

The results shown in Figure 3 make clear two points. First, since height information is not present in a d*I*/d*z* map we have to conclude that the surface is smooth and continuous. Second, d*I*/d*z* provides information on the apparent barrier height, which is a material property. No contrast is visible and therefore we have to conclude that we are dealing with the same material, i.e., MoS_2_. Both these points provide compelling evidence that the deposited Si intercalates between the MoS_2_ layers. For a comparable system, namely Si on WSe_2_, we recently arrived at a similar conclusion [45].

**Figure 4:**
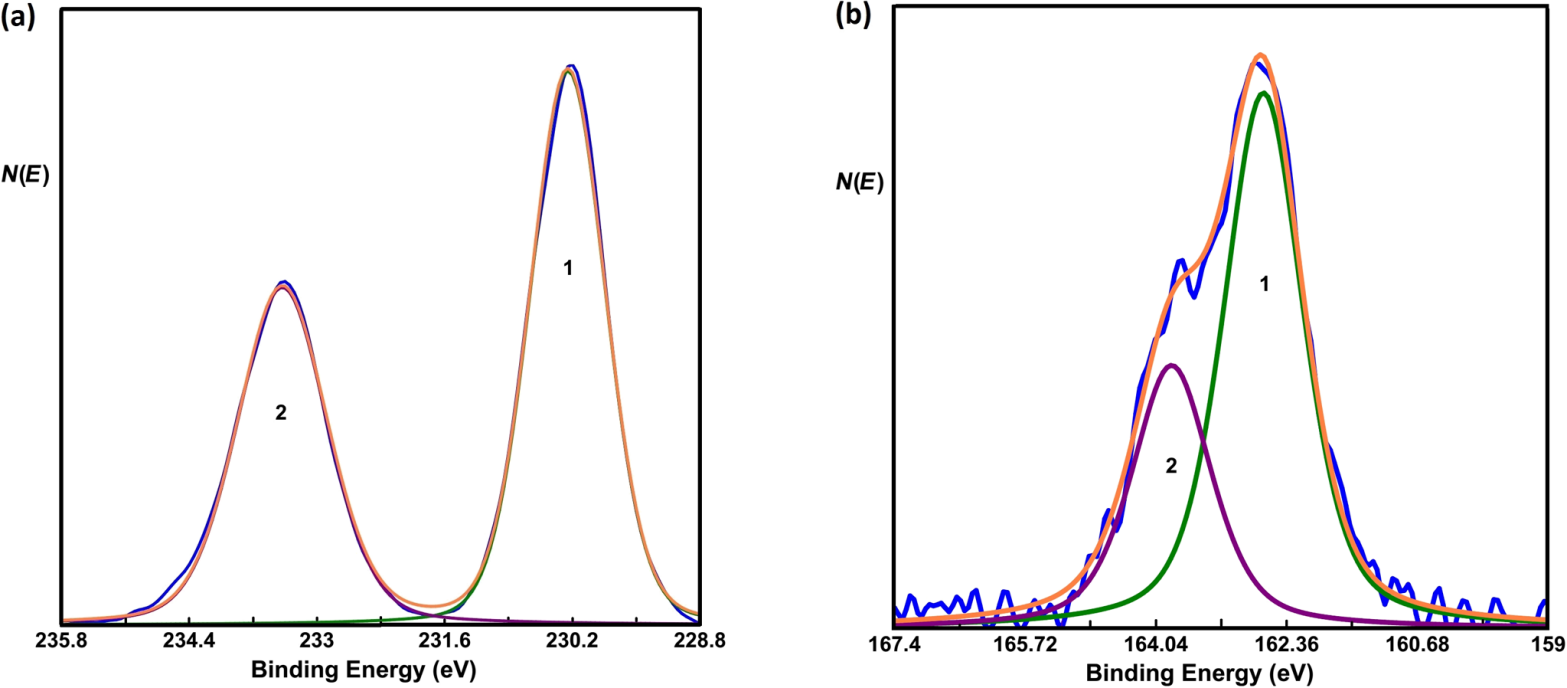
Core-level spectra of (a) Mo and (b) S before depositing Si. The spectra are fitted with two GL function peaks. In (a), 1 and 2 represent the Mo 3d_5/2_ and 3d_3/2_ peaks, respectively. In (b), 1 and 2 represent the S 2p_3/2_ and 2p_1/2_ peaks, respectively. In both figures, the resultant fitted spectra are represented by an orange line.

XPS measurements have been performed to obtain insight of the chemical composition of the top layers. Before depositing Si, XPS measurements were carried out on pristine MoS_2_ in order to find the exact positions of the Mo 3d_5/2_ and S 2p_3/2_ core-level peaks. (Figure 4a and Figure 4b, respectively). The Mo 3d_5/2_ and S 2p_3/2_ peaks were measured at 230.25 eV and 163.09 eV, respectively. The location of these peaks is in good agreement with [46–47].

**Figure 5:**
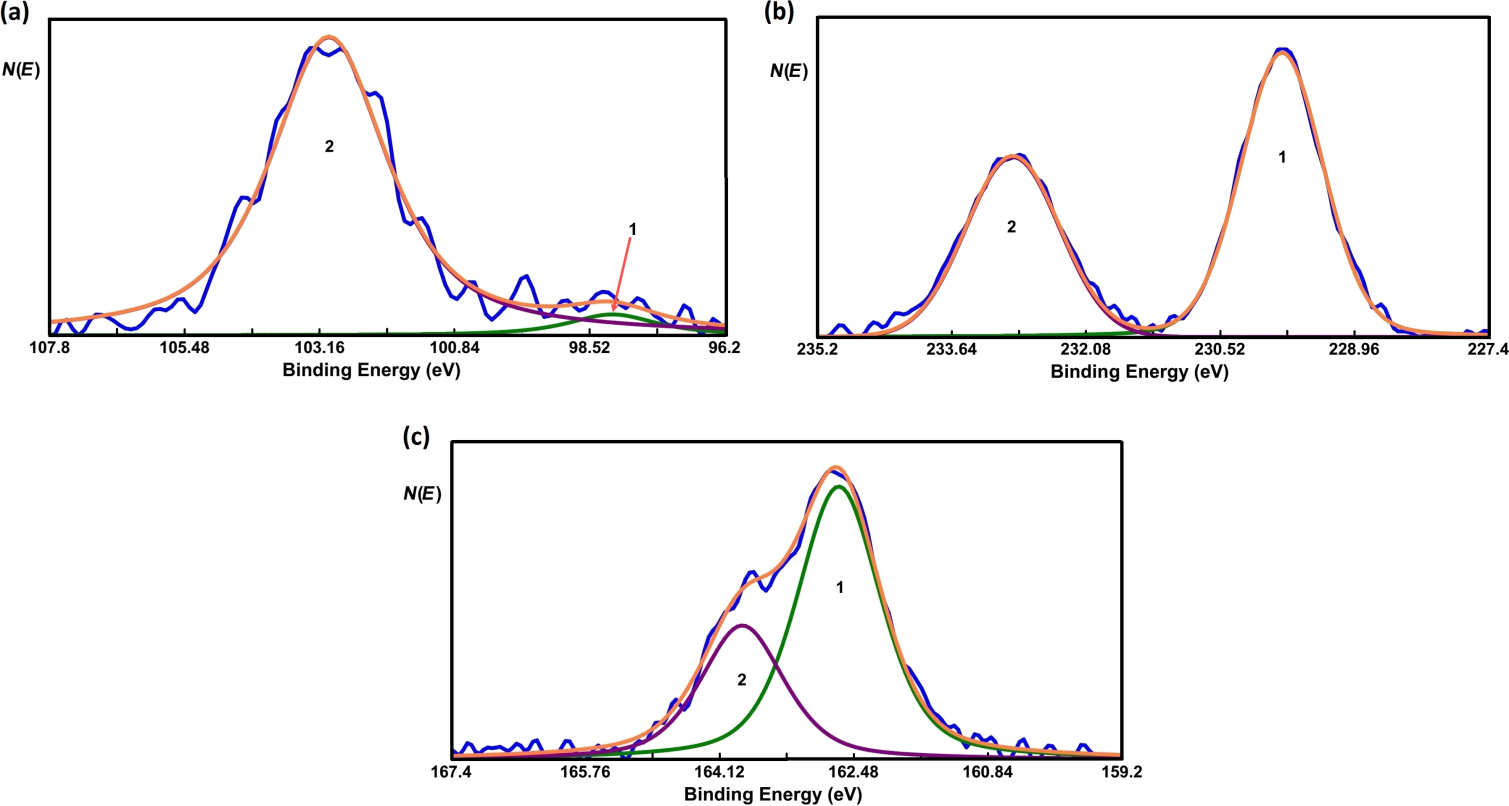
Core-level spectra of (a) Si, (b) Mo and (c) S after depositing Si. The peak-fitting procedure is the same as in Figure 4. In panel (a) a small peak at around 98.1 eV was needed to fit the tail of the peak at the lower-energy side. Orange lines represent the resultant fitted spectra.

The core-level spectra of Si, Mo and S after the deposition of 0.5 monolayers of Si on MoS_2_ are shown in Figure 5a, Figure 5b and Figure 5c, respectively. A higher coverage than in the case of STM is used in order to yield a stronger signal in the XPS measurements. STM topography images with a higher coverage can be found in Figure S1 in Supporting Information File 1. The XPS data show two peaks associated with Si. The smaller peak, located at 98.13 eV, can be attributed to pristine Si. The other peak, measured at 103 eV, can be attributed to oxidized silicon [48]. The oxidation of Si occurs during the transfer of the sample from the growth chamber to the XPS chamber. During this transfer the sample was exposed to ambient conditions. A more detailed analysis reveals that only 5% of the Si is pristine, whereas the rest is oxidized. Upon sputtering of the MoS_2_/Si sample with an Ar ion beam with 1 kV energy, we observe that the relative Si signal increases while the relative S signal decreases as can be seen in Figure S3 in Supporting Information File 1. This observation indicates that Si has intercalated between the MoS_2_ layers. In addition, we also conclude that the intercalated Si can be oxidized.

It is well known that numerous elements have a strong tendency to intercalate between MoS_2_ layers [49–50]. As for the intercalation mechanism of silicon in between MoS_2_ layers, we can only speculate. A plethora of studies on the intercalation of different chemical species in TMDs have been reported from elements as small as lithium [51], sodium [52–54] and carbon [55] to elements as large as cesium [56–57] and gold [58]. Other studies report on the intercalation of silicon and other elements under graphene layers synthesized on metal substrates [59–61].

The driving force for intercalation is charge transfer between the intercalated atoms and the layered material [62–63] or thermodynamic stabilization [61–62]. The mechanism of intercalation was found to occur through cracks and wrinkles in the layers [60] and via edges [51]. Because the diffusion barrier of adsorbed silicon atoms on top of MoS_2_ is assumed to be very low and the experiments are performed at room temperature, it is expected that silicon adatoms can easily diffuse over the surface to reach these cracks, wrinkles and step edges.

In order to study the effect of the oxidation of intercalated silicon in more detail we measured the exact positions of the Mo 3d_5/2_ and S 2p_3/2_ peaks. Both peaks shift to a lower binding energy by about 0.45 eV. This shift cannot be interpreted as a simple chemical shift due to a chemical reaction of the involved elements, i.e., Mo/S/Si and O [64]. In addition, after the deposition of Si no significant changes in the FWHM of the peaks of Mo (0.97 before, 1.13 after) as well as of S (1.09 before, 1.21 after) were observed, indicating that no chemical reaction between MoS_2_ and silicon oxide has occurred. It is very likely that the observed shift is attributed to a change in the position of the Fermi level.

It has been shown that the deposition of MoS_2_ on a SiO_2_ substrate with interface impurities leads to a charge transfer from the MoS_2_ surface to the defect states and, thus, to the formation of surface dipoles [65]. These dipoles shift the Fermi level of MoS_2_ closer to the valence band maximum (p-type). The shift of the Fermi level also leads to a shift in the binding energy of the Mo and S peaks to lower binding energies.

**Figure 6:**
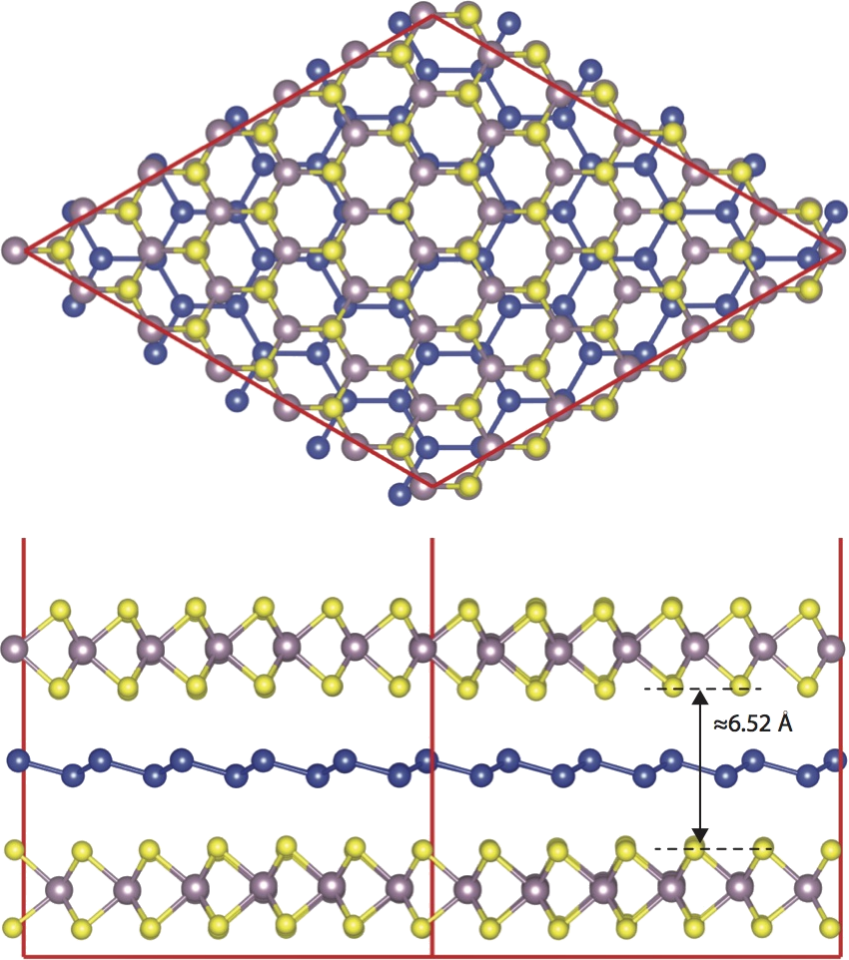
Top and side views of silicene intercalated in bilayer MoS_2_.

Next, we will discuss the results of our density functional theory calculations regarding the intercalated Si. To be consistent with experimental results we have fixed the lattice constant of MoS_2_ to 3.16 Å. We first calculated the structural and electronic properties of hetero-trilayers composed of a silicene layer intercalated between two MoS_2_ monolayers (MoS_2_–silicene–MoS_2_). Due to the large lattice mismatch, we have considered a commensurable supercell, in which we have placed a 5 × 5 silicene cell and a 6 × 6 MoS_2_ cell on top of each other. For this configuration the lattice mismatch of the MoS_2_–silicene–MoS_2_ trilayer becomes less than 1%. Figure 6 shows the optimized structure of the MoS_2_–silicene–MoS_2_ hetero-trilayer. The calculated interlayer distance in a pristine MoS_2_ bilayer is found to be 3.00 Å. Insertion of a silicene monolayer enlarges the interlayer separation between MoS_2_ layers from 3 Å to 6.52 Å, corresponding to an increase of the interlayer separation of 3.52 Å.

**Figure 7:**
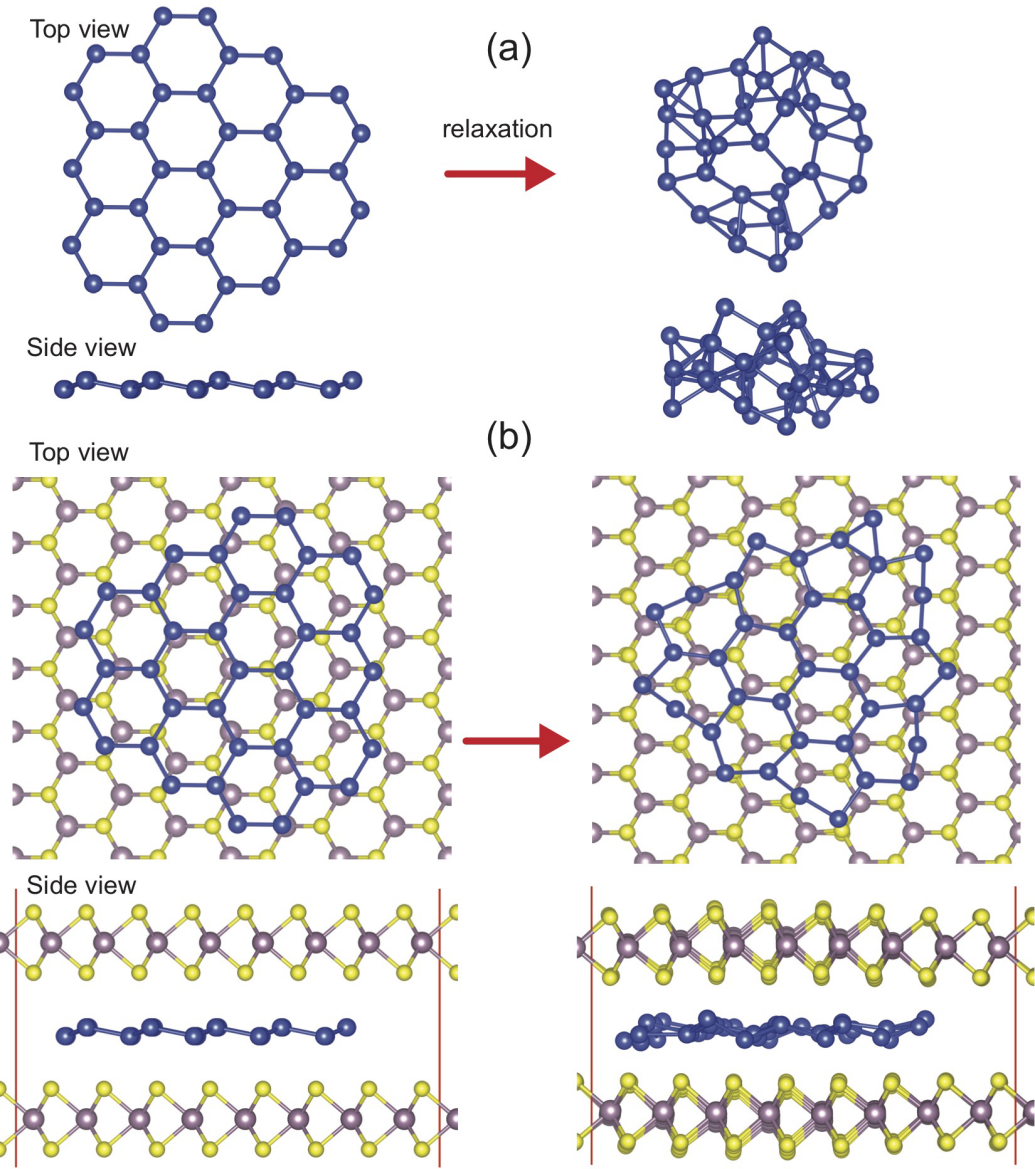
Initial (left) and equilibrium (right) structure of (a) a free standing and (b) an intercalated silicon cluster (Si_37_).

In order to study the possible formation of silicene between the MoS_2_ monolayers, we consider a buckled 2D silicon cluster (Si_37_) consisting of six-membered silicon rings. We used a 7 × 7 super-cell structure for the MoS_2_ bilayer. Initial and equilibrium geometries for both a free-standing as well as the intercalated silicon cluster inserted between the MoS_2_ layers are shown in Figure 7. We found that a free-standing 2D buckled silicon cluster is, in contrast to an infinite silicene layer, not even metastable in vacuum and spontaneously transforms into a strongly buckled 3D assembly as seen in Figure 7a. The intercalated silicon cluster in Figure 7b also undergoes a remarkable structural reconstruction. The optimized structure of a silicon cluster encapsulated between two MoS_2_ layers is totally different from the free-standing optimized silicon cluster in vacuum. This is noticeable in that the shape of the hexagons is not uniform as is the case for silicene. Especially at the edges, due to the presence of the Si dangling bonds, the hexagons are seriously distorted. However, intercalation between MoS_2_ layers preserves the 2D buckled structure of the silicon cluster during the structure relaxation. Thus, we suggest that the intercalation of silicon atoms between MoS_2_ layers may promote the formation of silicene, which interacts only weakly with the environment via van der Waals forces. We found that both top and bottom MoS_2_ layers develop bumps due to the interaction with the silicon cluster. The average interlayer MoS_2_ distance varies within the range of 5.5–6.2 Å, which corresponds to an increase in interlayer separation of 2.5-3.2 Å. This agrees well with the measured height variation.

## Conclusion

In this work we revisited the growth of Si on MoS_2_. STM topography data reveals that Si does not grow on top of the MoS_2_ substrate, but rather intercalates in between the MoS_2_ layers. It is known that layered materials such as MoS_2_ have a tendency to host intercalants. In this work we provide additional evidence for silicon intercalation by using STS and XPS. Since silicon intercalates it is interesting to scrutinize if there are possibilities to grow a 2D layer in between two layers of MoS_2_. Our density functional theory calculations show that 2D silicon clusters intercalated between MoS_2_ layers are stable.

## Supporting Information

File 1 Additional experimental data.
