# Supplementary material for: Intercalation of Si between MoS2 layers
Source: Beilstein J Nanotechnol. 2017 Sep 19;8:1952–60. doi: 10.3762/bjnano.8.196 (PMC5629401; doi:10.3762/bjnano.8.196)
Supplement: File 1 — Additional experimental data. [file Beilstein_J_Nanotechnol-08-1952-s001.pdf]

Supporting Information

for

Intercalation of Si between MoS_2 layers

Rik van Bremen[‡,1], Qirong Yao[‡,1], Soumya Banerjee[2], Deniz Cakir[2], Nuri Oncel[2] and Harold J. W. Zandvliet[*,1]

Address: [1]Physics of Interfaces and Nanomaterials, MESA[+] Institute for Nanotechnology, University of Twente, P.O. Box 217, 7500AE Enschede, Netherlands and [2]Department of Physics and Astrophysics, University of North Dakota, Grand Forks, ND 58202, USA

Email: Harold J. W. Zandvliet* - h.j.w.zandvliet@utwente.nl

* Corresponding author

‡ Equal contributors

Additional experimental data

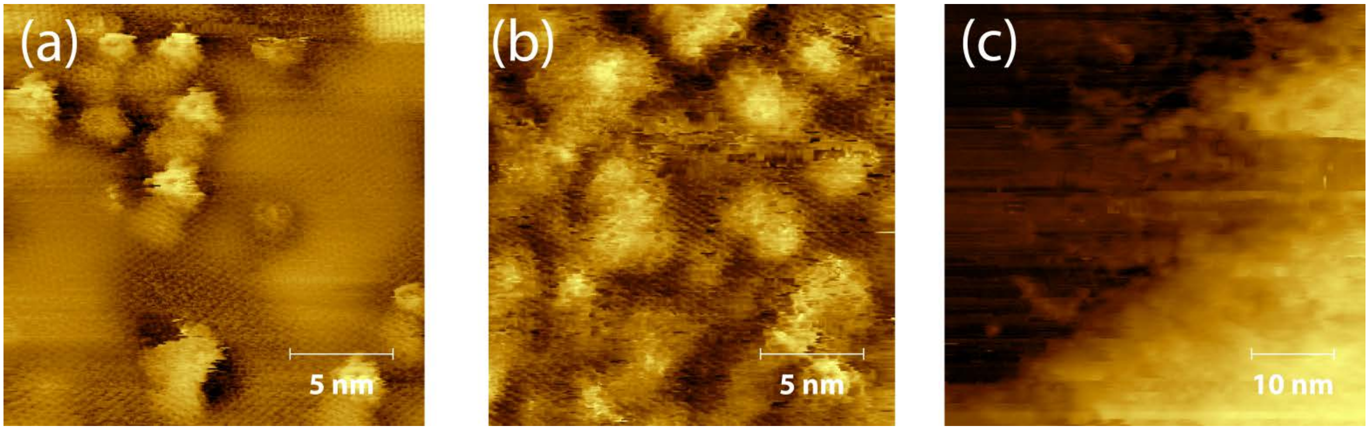

Figure S1: Topography of the MoS_2 surface as a function of the amount of deposited silicon. (a) 0.2 monolayers. Here transitions between hills and valleys are visible. The recurring feature is a tip image. (b) 0.8 monolayers. The sample becomes rougher and more difficult to image. (c) 1.4 monolayers. Silicon clusters are visible on top of the MoS_2.

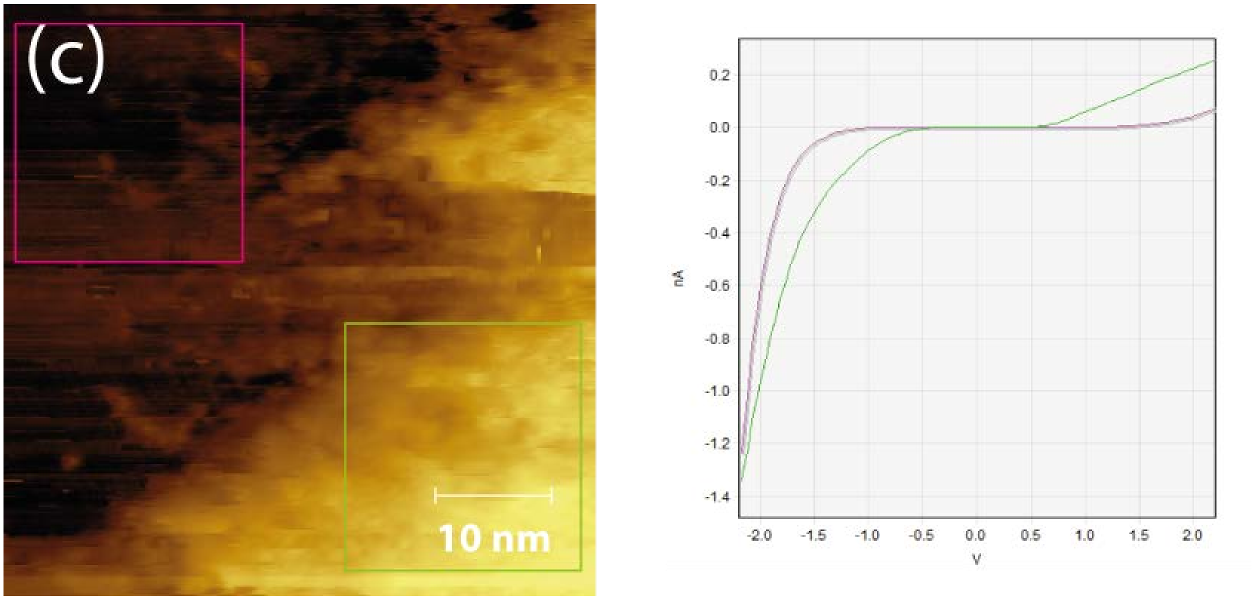

Figure S2: (a) silicon clusters on top of MoS_2 after deposition of 1.4 monolayers of silicon. The purple and green boxes indicate the locations where a grid scan is performed. Each grid did consist of 100 curves. (b) The average of the recorded $I(V)$ curves on top of the cluster (green) is significantly different from the average of the curves recorded on MoS_2 (purple).

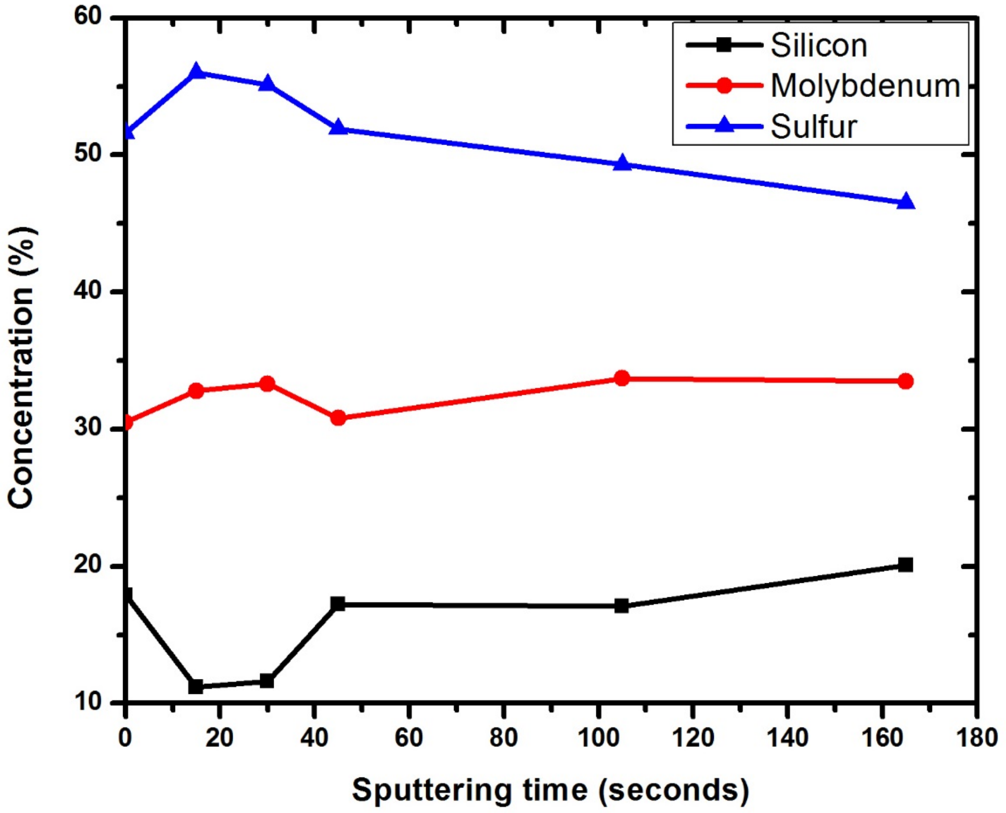

Figure S3: Atomic concentration of Si, Mo and S as a function of the sputtering time. The sputter current used is 0.33 µA with an estimated spot diameter of 2 mm.
